# Effective Dysphonia Detection Using Feature Dimension Reduction and Kernel Density Estimation for Patients with Parkinson’s Disease

**DOI:** 10.1371/journal.pone.0088825

**Published:** 2014-02-20

**Authors:** Shanshan Yang, Fang Zheng, Xin Luo, Suxian Cai, Yunfeng Wu, Kaizhi Liu, Meihong Wu, Jian Chen, Sridhar Krishnan

**Affiliations:** 1 School of Information Science and Technology, Xiamen University, Xiamen, Fujian, China; 2 Department of Rehabilitation, Zhongshan Hospital Xiamen University, Xiamen, Fujian, China; 3 Department of Electrical and Computer Engineering, Ryerson University, Toronto, Ontario, Canada; The University of Chicago, United States of America

## Abstract

Detection of dysphonia is useful for monitoring the progression of phonatory impairment for patients with Parkinson’s disease (PD), and also helps assess the disease severity. This paper describes the statistical pattern analysis methods to study different vocal measurements of sustained phonations. The feature dimension reduction procedure was implemented by using the sequential forward selection (SFS) and kernel principal component analysis (KPCA) methods. Four selected vocal measures were projected by the KPCA onto the bivariate feature space, in which the class-conditional feature densities can be approximated with the nonparametric kernel density estimation technique. In the vocal pattern classification experiments, Fisher’s linear discriminant analysis (FLDA) was applied to perform the linear classification of voice records for healthy control subjects and PD patients, and the maximum *a posteriori* (MAP) decision rule and support vector machine (SVM) with radial basis function kernels were employed for the nonlinear classification tasks. Based on the KPCA-mapped feature densities, the MAP classifier successfully distinguished 91.8% voice records, with a sensitivity rate of 0.986, a specificity rate of 0.708, and an area value of 0.94 under the receiver operating characteristic (ROC) curve. The diagnostic performance provided by the MAP classifier was superior to those of the FLDA and SVM classifiers. In addition, the classification results indicated that gender is insensitive to dysphonia detection, and the sustained phonations of PD patients with minimal functional disability are more difficult to be correctly identified.

## Introduction

Dysphonia is a type of phonation disorder with an impairment in the ability to produce normal voice sounds [1]. Manifestation of dysphonic voice is characterized by hoarseness or weakness in phonation [2]. As the functional causes of dysphonia, neurological disorders sometimes make neurogenic interruptions in the laryngeal nerve paths that could interfere in normal vibration of vocal folds during exhalation [3]. Dysphonia is detrimental to quality of life, because the speech impaired patient often encounters difficulty in personal communication that leads to depression and further social handicap [4]. A large number of patients with idiopathic Parkinson’s disease (PD) suffer from dysprosody and dysarthria [5]. According to the survey of Hartelius and Svensson [6], over 70% of the PD patients experienced speech deficit and voice impairment after the onset of their disease, and only 3% of the patients had received speech therapy. Ho et al. [5] utilized the clinical-perceptual method to study the speech difficulties in PD. They sampled the two-minute conversational speech of 200 PD patients, and examined the speech deficit profiles (i.e., voice, articulation, and fluency) [5]. Their study showed that voice was the leading deficit in the initial stage of PD, and articulatory and fluency deficits manifested in the severe stage of PD [5].

Quantitative measures of speech impairment could help assess the severity levels of speech impairment in PD patients and study the specific impaired speech parameters [7]. The simultaneous qualitative and quantitative investigations are able to characterize the exaggerated vocal tremor, weak voice, roughness, and other dysphonic symptoms in idiopathic PD, which sometimes would be confused with spasmodic dysphonia (a laryngeal abnormality characterized by spasm of the vocal cord) in clinical diagnosis [8]. Recently, telemedicine systems with advanced network access have been effectively used for remote monitoring of patients with vocal impairment [9]. The telemedicine technology provides relatively low-cost clinical monitoring solutions that help reduce frequent physical visits for patients [10]. As suggested by Little et al. [10], such telemedicine systems call for more reliable clinical tools and speech measurements for accurate detection and monitoring of vocal symptoms in PD.

A number of novel speech measurement methods have been developed to assess dysphonic symptoms in the last decade [2], [3], [11]–[13]. The purpose of such speech measurements is to characterize the features of the acoustic signals associated with phonation disorders. Impairment of vocal folds often causes the irregular movement in one or both sides of the glottis that leads to pathological vibration patterns, such as pitch frequency fluctuations, changes of airflow volume, and amplitude alteration [2]. Thus, dysphonia is often observed in the production of vowel sounds. The fundamental frequency (F0) in vowels, mean of F0, variation of F0 (jitter), the variation of speech amplitude (shimmer), intensity from one vocal cycle to another are the most frequently used electroglottographic measures in standard speech tests [14]–[17]. Zwirner and Barnes [14] reported that the standard deviation of F0 in prolonged vowels is much larger for PD patients compared with healthy control subjects. The study of Hertrich and Ackermann [15] indicated increased jitter and higher mean of F0 in prolonged vowels for PD patients. Goberman et al. [16], [17] compared the acoustic-phonatory characteristics of Parkinsonian speakers before and after taking medication, and found greater F0 variability and decreased intensity range (difference between the loudest and quietest prolongations) in the disfluent speech of PD patients. Cnockaert et al. [18] used the wavelet analysis technique to extract the phonatory frequency trace and low-frequency vocal modulation in sustained vowels. Their study suggested that the average phonatory frequency is significantly higher for male subjects with PD, and the modulation amplitude is significantly larger for female PD patients [18]. Recently, nonlinear dynamics analysis tools have been utilized to study the vocal oscillation patterns in Parkinsonian phonatory impairment. Rahn et al. [19] employed the phase space reconstruction and correlation dimension methods to measure the perturbation nature in the aperiodic voices of idiopathic PD. Their results showed that the correlation dimension values are significantly higher in PD patients than those of control subjects, which implies an increased complexity of phonatory signals in PD vocal pathology [19]. In order to overcome the limitations of vocal perturbation measurement in the time scale and Fourier transform in the frequency domain, Little et al. [13] proposed the time-delay state-space recurrence analysis and the fractal scaling analysis to exploit the nonlinearity in pathological phonation voices. The detrended fluctuation analysis and quadratic discriminant analysis methods were used to investigate the self-similarity properties (in terms of scaling exponent) of vocal fluctuations associated with PD [10], [13]. In the present work, we aim to study the mutual correlations among the different parameters in vocal measurements, and also to develop an effective method for the vocal pattern classification. It is hypothesized that the most informative features with regard to fundamental frequency, amplitude variability, and dynamics in vocal fluctuations could be properly selected and used in the nonlinear analysis methods for the accurate classification of PD patterns.

## Materials and Methods

### Dataset Preparation

**Figure 1:**
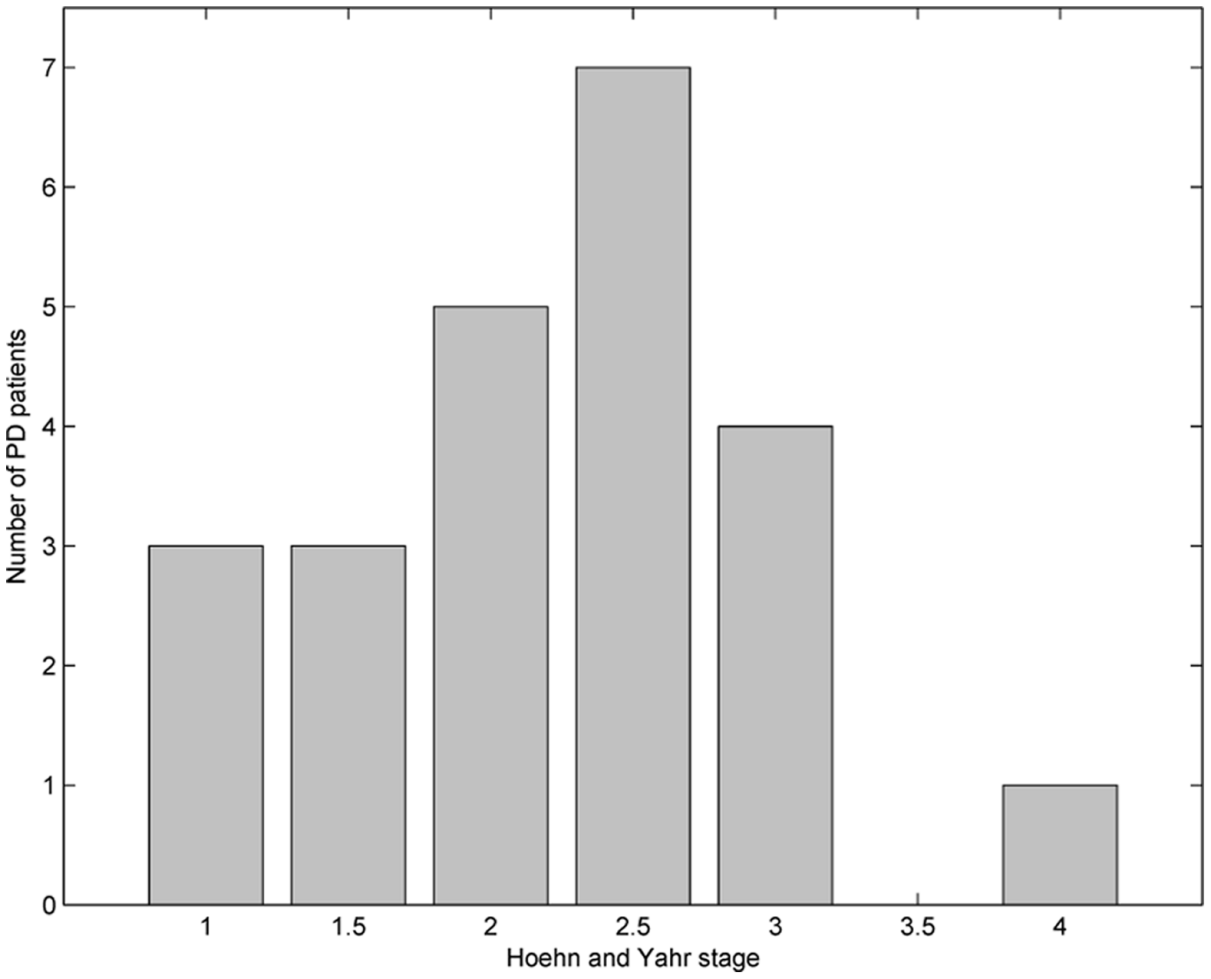
Severity of neurological impairment in terms of Hoehn and Yahr (H&Y) scale for the patients with Parkinson’s disease (PD).

The data set used in this study was donated by Little et al. [10], and is also online available via University of California at Irvine (UCI) machine learning repository [20]. The phonation data contain 195 sustained vowel records uttered by total 31 subjects. There were 8 healthy control subjects (3 males and 5 females), with the averaged age of 60.2 years (standard deviation: 8.6 years), participating in the speech tests. The PD patients included 16 males and 7 females (mean and standard deviation of age: 67.8±9.7 years). The disease stage of each PD patient was assessed with the Hoehn and Yahr (H&Y) scale [21], a widely used PD progression rating method in clinical practice. Figure 1 shows the detailed numbers of the PD patients with different H&Y stages. It can be observed that majority (82.6%) of the PD patients underwent the intermediate course of the disease (1<H&Y<3.5).

The vocal vowels were recorded using a head-mounted microphone positioned at 8 cm from the lips. The microphone was calibrated with a Class 1 sound level meter (Brüel & Kjær Type 2238 Mediator) placed 30 cm in front of each subject [10]. The acoustic signals were digitized with the resolution of 16 bit and the sampling rate of 44.1 kHz, and the signal samples were normalized in amplitude [10]. Little et al. [10] implemented the Kay Pentax multidimensional voice program (MDVP) to measure the perturbations in the sustained vowel records. Such perturbation measures include the period (jitter) and amplitude (shimmer) perturbations and harmonics-to-noise (and noise-to-harmonics) ratios. They also computed the nonlinear parameters such as correlation dimension (D2), recurrence period density entropy (RPDE), detrended fluctuation analysis (DFA), and pitch period entropy (PPE) [10]. Based on the vocal perturbation and nonlinear measures, the standard support vector machine (SVM) was applied to distinguish the normal and pathological vocal patterns [10]. The data set also contained two nonlinear measures of fundamental frequency variation: Spread1 and Spread2. All the subjects recruited in the experiments of Little et al. [10] provide their written informed consent as supervised by University of Oxford, United Kingdom, and U.S. National Center for Voice and Speech, Denver, Colorado. The data analysis methodology documents of this study were approved by the Institutional Review Board of Xiamen University.

### Feature Analysis

#### Feature dimension reduction

**Figure 2:**
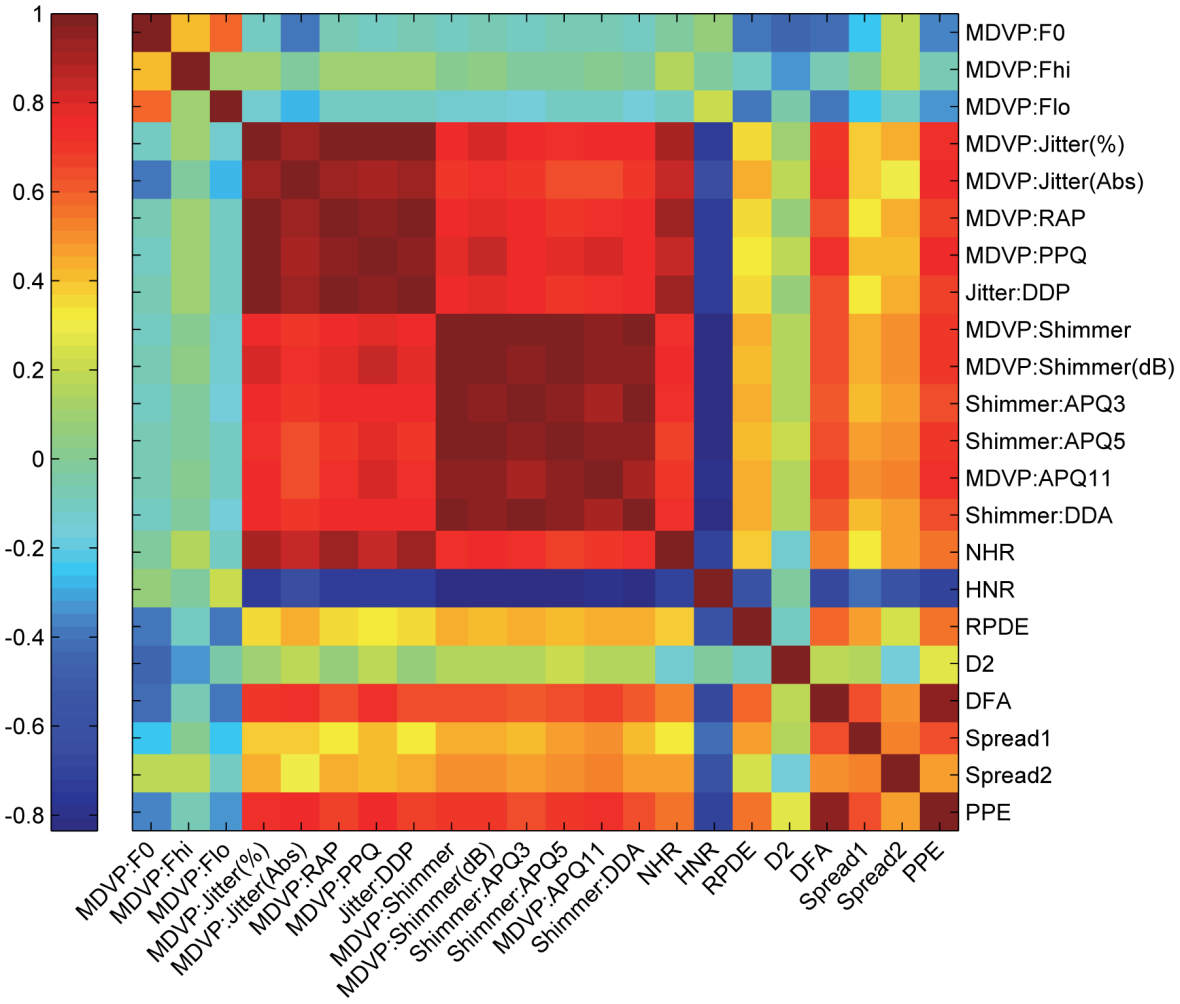
Graph visualization of correlation matrix for the vocal measurements, with the color map ranging from blue (for negative correlation coefficient) to red (for positive correlation coefficient).

The correlation matrix between pairs of vocal measures is shown in Fig. 2. It is noted that a number of vocal measures are highly correlated with the others, because some measures indicated the similar characteristics of the acoustic signals in the data set. The similar vocal measure groups are period perturbations (MDVP: Jitter (%), MDVP: Jitter (Abs), MDVP: RAP, MDVP: PPQ, Jitter: DDP), amplitude perturbations (MDVP: Shimmer, MDVP: Shimmer (dB), Shimmer: APQ3, Shimmer: APQ5, MDVP: APQ11, Shimmer: DDA), and nonlinear measures (DFA, PPE). In particular, the Shimmer: DDA and Shimmer: APQ3 measures exhibit completely collinear relationship (the correlation coefficient equal to 1). Little et al. [10] first normalized the feature values in the numerical range [−1, 1] to ameliorate the classification performance of support vector machine (SVM). Then they searched through the pairs of highly correlated measures (the correlation coefficient larger than 0.95), and removed an arbitrary measure in each pair [10]. The correlation filtering procedure excluded the following measures: MDVP: Jitter (%), MDVP: RAP, MDVP: PPQ, MDVP: Shimmer, MDVP: Shimmer (dB), Shimmer: APQ3, Shimmer: APQ5, with the remaining ten measures for further SVM classification [10].

In this investigation, we performed the sequential forward selection (SFS) method [22] to select the dominant measures and exclude the similar measures that contribute the redundant information. The logistic regression [23] was employed in the SFS selection process to evaluate the performance. The SFS is a greedy searching algorithm that starts from an empty feature set, and then sequentially adds and combines with the features to maximize the logistic regression performance. The feature set obtained by the SFS method included: MDVP: F0, MDVP: Jitter (%), DFA, Spread2.

We also implemented the kernel principal component analysis (KPCA) to project the SFS features onto the two-dimensional mapping space. The PCA makes the orthogonal transformation to convert multivariate measures into some linearly uncorrelated principal components. The KPCA is a kernel-based extension of PCA method that conducts a nonlinear feature mapping in the kernel Hilbert space [24]. In the present work, the KPCA was performed with the polynomial kernel which can be expressed as [23]:
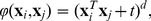
(1)where 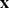 represents the vector of SFS-selected vocal measures, 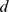 denotes the polynomial order, and 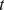 is the intercept. We searched the polynomial kernel parameters in the range from 1 to 10, and chose 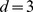 and 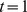, which could make a maximum Euclidean distance of vocal patterns between the healthy control and PD subject groups.

#### Feature density estimation

In the present work, we used the nonparametric kernel density estimation technique in order to provide the vocal pattern distribution of the KPCA-mapped features. The principle of the kernel density estimation uses the finite observed pattern scatters to approximate the nature of class distributions. Let the vector 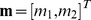 denote the KPCA feature set, where 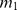 and 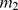 are used to express the first and second principal components. The class label of the vocal pattern is represented as 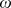, with 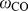 and 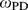 denoting the groups of healthy controls and PD patients, respectively. Based on the 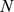 vocal patterns from a particular subject group, the kernel density estimation method can approximate the class-conditional feature density with kernel functions as [25]

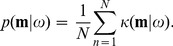
(2)


The bivariate Gaussian kernel function is presented as
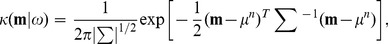
(3)where 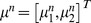 indicates the center location of the 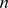-th vocal pattern in the subject group 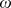.

Because the KPCA produces the orthogonal projection for the principal components, the correlation coefficient between the first and second principal components is equal to zero. Hence, the covariance matrix that determines the spread width of the Gaussian kernel function is a diagonal matrix, written as 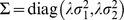, in which 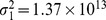 and 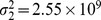 are the variances of first and second principal components, respectively. The scaling factor 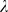 is used to coordinate the spread area of the Gaussian kernel function in the KPCA feature space. We searched the scaling factor in the numerical range [0, 1] with an increment of 0.01, and selected 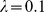 that could make the best contour resolution of estimated KPCA feature densities in the present study. Regulated by the scaling factor parameter 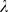, the 2-by-2 diagonal covariance matrix became 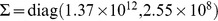. In the computer experiment, the covariance matrix was unique for both groups of healthy controls and PD patients.

### Vocal Pattern Classification

With the estimated class-conditional densities of KPCA features, we employed the maximum *a posteriori* (MAP) rule (also referred to as Bayes decision rule) [26], to perform the classification of vocal patterns. In the present work, *a posteriori* probability 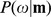 indicates the possibility of a vocal pattern with its observed feature vector 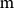 belonging to either healthy control or PD voice record group 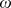. According to Bayes formula, *a posteriori* probability 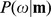 can be computed from the class-conditional probability density 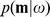 as
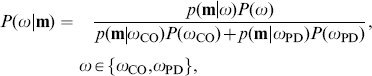
(4)where the class-dependent *a priori* probability 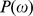 presents the possible occurrence of a particular vocal record group. The MAP classifier recognizes the observed vocal pattern 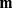 belonging to the PD record if its *a posteriori* probability 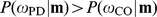, vice versa.

In addition to the MAP classifier, we also implemented the SVM for classification performance comparison. The SVM is a kernel-based artificial neural network, which trains the network parameters to minimize the structural risk [27]. The SVM is able to perform the same function as the multilayer neural network (such as multilayer perceptron or radial basis function network), by choosing the corresponding nonlinear inner-product kernels. During the SVM parameter optimization, the training data which geometrically locate close to the decision boundary will be selected as the support vectors, which are considered to be informative for the classification. The SVM learning can be formulated as the following constrained quadratic programming problem with respect to the convex cost function [27]–[29]:
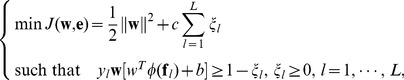
(5)where 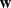 and 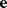 are the weight and error vectors, 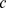 is a positive real constant, and 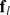 denotes the KPCA-mapped feature vector of the 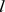-th vocal pattern. In the present work, we compared the performance of the SVM with polynomial, sigmoid, and radial basis function (RBF) kernels, and then chose the standard RBF kernels 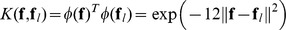 to construct the support vectors.

We also employed the Fisher’s linear discriminant analysis (FLDA) to perform the binary classification of vocal patterns. The FLDA does not require the assumptions that the patterns of different groups are with the normal distributions or equal class covariances. The FLDA searches the parameter vector 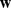 that maximizes the class separability in the feature space to perform the linear discriminant as [23], [30]

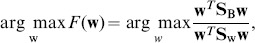
(6)where 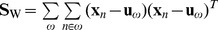 is the within-class scatter matrix as the sum of intra-class variances, and 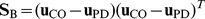 is the between-class scatter matrix.

In the present study, we implemented the 5-fold cross-validation method to evaluate the classification performance for each classifiers. The whole data set was partitioned into five disjoint subsets. Four subsets were used to train the classifiers, and the remaining subset was used for testing. The procedure was repeated for five trials, each time using a different subset for validation.

## Results

The SFS method selects the MDVP: F0, MDVP: Jitter (%), DFA, and Spread2 as dominant measurements. It can be observed from Table 1 that four vocal measurements possess different metric range. For example, the difference of mean values between CO and PD records for MDVP: F0 is with a much larger order of magnitudes than that for MDVP: Jitter (%), although both of these two measurements present the vocal perturbations in fundamental frequency. In previous work of of Little et al. [10], all of these vocal measurements were normalized to diminish the influence of variant measurement magnitudes upon further classifications. In our experiments, the KPCA was applied to reduce the feature dimensions by projecting four vocal measurements onto a bivariate space, in which the vocal patterns with the KPCA-mapped features also exhibit distinct scatter distributions.

**Table 1:** Statistics of the vocal measurements selected by the sequential forward selection (SFS) method.

Vocal measurements	Mean ± Standard Deviation	
	CO records	PD records
MDVP: F0 (Hz)	181.938±52.731	145.181±32.348
MDVP: Jitter (%)	0.004±0.002	0.007±0.005
DFA	0.696±0.051	0.725±0.055
Spread2	0.16±0.063	0.248±0.078

CO: healthy controls; PD: Parkinson’s disease; DFA: detrended fractal analysis.

**Figure 3:**
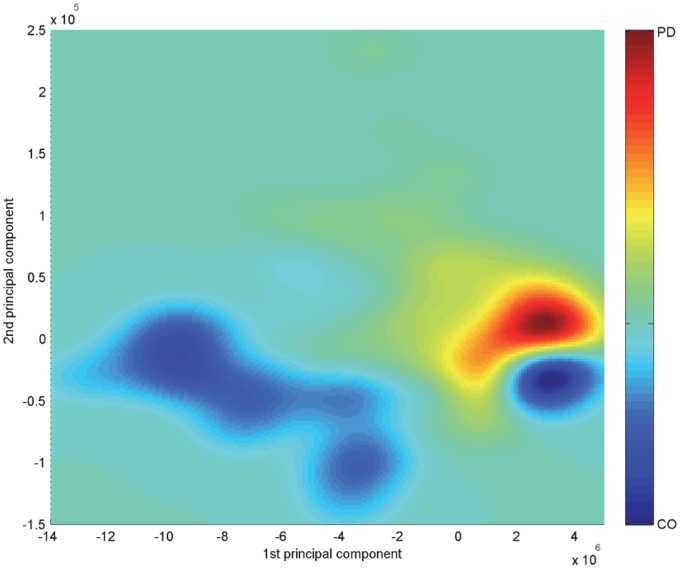
Bivariate distributions of vocal patterns in the kernel principal component analysis (KPCA) mapping feature plane. Vocal pattern distributions for the healthy controls (CO) and patients with Parkinson’s disease (PD) are displayed with the cold color map (blue for the highest density) and hot color map (red for the highest density), respectively.

**Figure 4:**
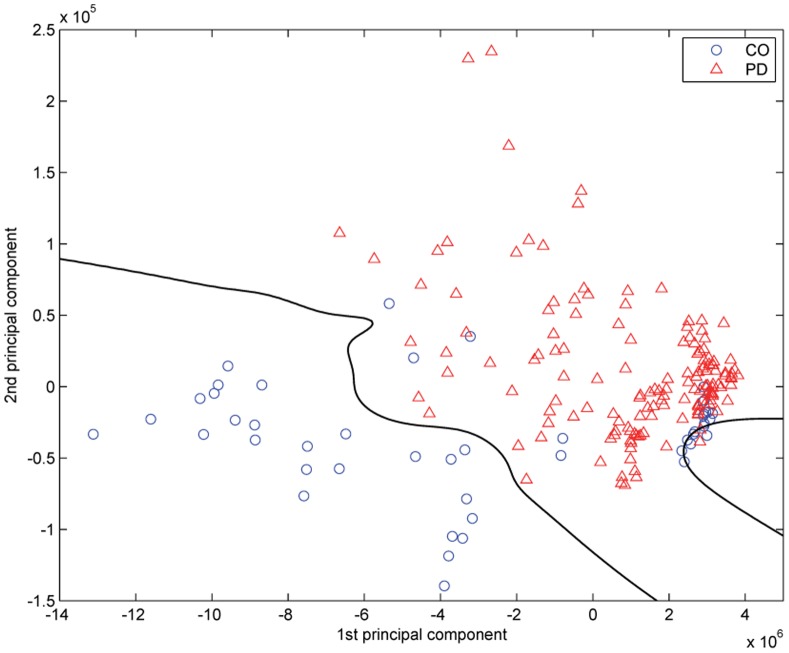
Scatter plots of the vocal patterns in the first and second kernel-based principal components plane for healthy controls (CO) and patients with Parkinson’s disease (PD). The decision boundary provided by the maximum *a posteriori* (MAP) classifier separates the vocal patterns associated with the CO and PD groups.

Figure 3 provides the estimated vocal pattern densities of CO and PD groups in the KPCA-mapped feature space. In accordance with the scatters located in Fig. 4, the aggregation area of vocal patterns associated with PD patients shows a high density in red. On the other hand, the vocal patterns of CO group possess multimodal density characteristics. As depicted in Fig. 4, majority of CO vocal patterns (30 records) disperses at the bottom left side of the feature space. In addition, about a quarter number of CO vocal patterns (18 records) converges as a small cluster in the lower right corner (see the high density area in blue color). The estimated feature densities make the vocal pattern distribution visible in the bivariate space.

**Figure 5:**
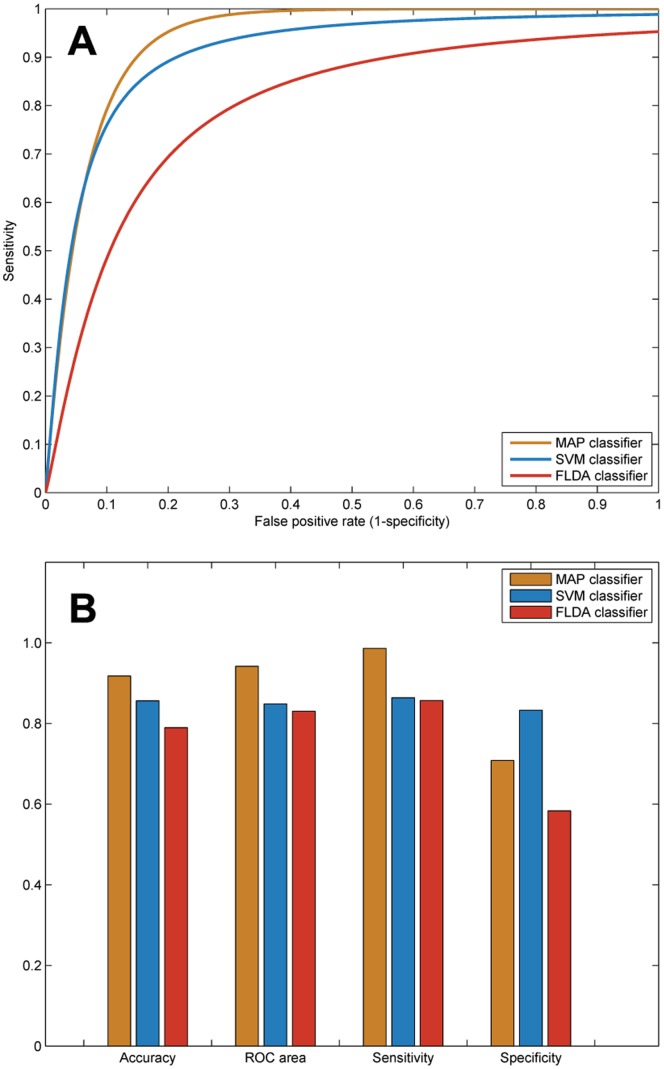
Diagnostic performance of the classifiers: (**A**) receiver operating characteristic (ROC) curves produced by the maximum *a posteriori* (MAP) classifier, support vector machine (SVM), and Fisher’s linear discriminant analysis (FLDA); (**B**) results of classification accuracy, sensitivity, specificity, and area under receiver operating characteristic (ROC) curve obtained by the three classifiers.

Figure 5 plots the classification results obtained by the three classifiers. The MAP classifier successfully distinguishes 91.8% (179 voice records among the total 195 records) vocal patterns, with a sensitivity rate of 0.986 (145 correct PD records), a specificity rate of 0.708 (34 correct CO records), and an area of 0.94 under the receiver operating characteristic (ROC) curve. There are 167 voice records correctly distinguished by the SVM (overall accuracy: 85.6%, ROC area: 0.85), including 127 PD records (sensitivity: 0.864) and 40 CO records (specificity: 0.833). The FLDA provides a linear classification with the accurate rate of 79% (154 correct voice records), sensitivity rate of 0.857 (126 PD records), specificity rate of 0.583 (28 CO records), and an area of 0.83 under ROC curve.

It is worth noting from Fig. 5B that the MAP classifier outperforms the other two classifiers with higher degrees of accuracy, area under ROC curve, and sensitivity. Such results imply that the MAP classifier has the superiority in recognition of PD voice records over the SVM and FLDA. On the other hand, the SVM classifier produces a higher specificity rate than either the MAP or FLDA, which indicates some merits for categorization of CO voice records. In general, the nonlinear classification (by means of the MAP or the SVM) is better than the linear classification (by means of the FLDA). The FLDA does not achieve the results obtained by either of the nonlinear classifiers, in any of classification evaluation criteria (i.e., accuracy, area under ROC curve, specificity, and specificity).

Table 2 lists the detailed subject information related to the voice records misclassified by the SVM and MAP classifiers. Only one PD patient’s speech was not accurately identified by the MAP classifier. Noting that the PD patient is with minimal functional disability (with H&Y stage 1), such a misclassification could be tolerated in clinical applications. A number of the voice records misclassified by the MAP classifier were spoken by the subjects S43, S49, and S50. Someone may suppose whether the MAP classification tends to be subject-dependent. But we observe that the same records are misclassified by the SVM classifier too. Such results, in our opinion, are not persuadable to infer that the MAP classification is subject-dependent. For the SVM classification, on the other hand, the voice records of more subjects are not correctly detected. Some subjects are with mild to moderate disability (with H&Y stages 2 through 3), which implies the weakness of the SVM classifier for pathological voice detection. We assume that the limited size of data set could be one possible cause. The SVM classifier needs to select informative support vectors to construct the decision boundary, therefore a small number of phonation data would result in a bias of the decision making. In addition, the number of male subjects is not significantly different from that of female subjects in either of two misclassification lists, which indicates that the gender is insensitive in the pathological voice detection for Parkinson’s disease.

**Table 2:** Details of subject information on the records misclassified by the maximum *a posteriori* (MAP) and support vector machine (SVM) classifiers.

MAP						SVM					
Subject ID	Failure records	Gender	Age	Group	H&Y stage	Subject ID	Failure records	Gender	Age	Group	H&Y stage
S32	2	M	50	PD	1.0	S02	1	M	60	PD	2.0
S42	1	F	66	CO	N/A	S04	1	M	70	PD	2.5
S43	6	M	62	CO	N/A	S08	1	F	48	PD	2.0
S49	4	M	69	CO	N/A	S19	1	M	73	PD	1.0
S50	3	F	66	CO	N/A	S20	1	M	70	PD	3.0
						S25	2	M	74	PD	3.0
						S26	3	F	53	PD	2.0
						S27	1	M	72	PD	2.5
						S32	5	M	50	PD	1.0
						S42	1	F	66	CO	N/A
						S49	3	M	69	CO	N/A
						S50	3	F	66	CO	N/A

M: male; F: female; CO: healthy controls; PD: Parkinson’s disease; H&Y stage: Hoehn and Yahr stage; N/A: not applicable for healthy subjects.

## Discussion

The selected features: MDVP: F0, MDVP: Jitter (%), DFA, and Spread2 provided the useful information about pathological voice in different clinical aspects. The fundamental frequency F0 quantifies the vibration frequency of the vocal folds. The period perturbation measure jitter corresponds to the cycle-to-cycle variation in fundamental frequency. The interruptions caused by Parkinson’s disease in the nervous paths could result in neurogenic paralysis of the recurrent laryngeal nerves, the superior laryngeal nerves, or the vagus nerves. The irregular vibration of the vocal folds would change the mean of F0, frequency variability (jitter), and speech amplitude, which could be measured in the phonation course of a sustained vowel. On the other hand, the DFA is used to describe the stochastic self-similarity properties of the noise caused by turbulent airflow in the vocal tract. Breathiness and other dysphonic voice caused by incomplete vocal fold closure would lead to an increase of the DFA value [9]. The nonlinear dynamical complexity parameter Spread2 can also characterize the extent of turbulent noise in the acoustic signal [10]. The Spread2 value is quite strongly associated with the dysphonia response. The present study demonstrated the predominant contributions of these four features to the analysis of PD vocal patterns.

**Figure 6:**
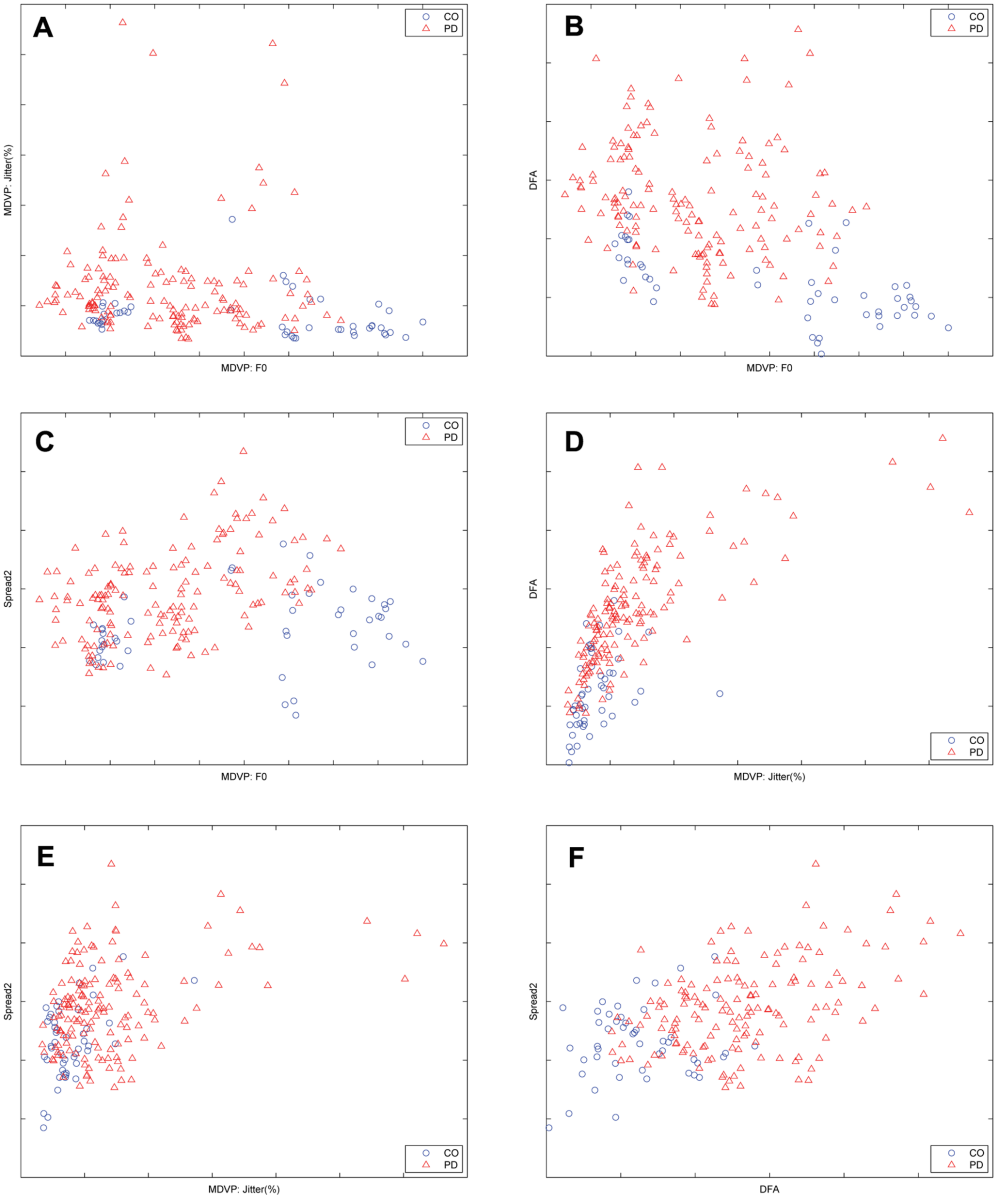
Scatter plots of the vocal patterns associated with the healthy controls (CO) and patients with Parkinson’s disease (PD) in the two-dimensional feature spaces of (**A**) MDVP: F0 and MDVP: Jitter (%), (**B**) MDVP: F0 and detrended fluctuation analysis (DFA), (**C**) MDVP: F0 and Spread2, (**D**) MDVP: Jitter (%) and DFA, (**E**) MDVP: Jitter (%) and Spread2, and (**F**) DFA and Spread2, respectively.

Figure 6 plots the scattered vocal patterns with pairs of the selected features. It is worth noting that the vocal patterns associated with the healthy controls and PD patients are still overlapping in the two-dimensional feature spaces. Among these combinations of the selected feature pairs, the feature pairs of MDVP: F0–DFA and DFA–Spread2 could provide relatively better separable pattern distributions in Fig. 6B and 6F. We validated the performance of the MAP classifier with these two feature pairs, using the 5-fold cross-validation method. The accurate classification rates were 85.1% (ROC area: 0.9) and 85.6% (ROC area: 0.93) for the MDVP: F0–DFA and DFA–Spread2 feature pairs respectively, which were worse than the results obtained with the KPCA-based features. It is clear that the KPCA method can project the selected vocal features, with the nonlinear kernels, onto the visible bivariate space toward superior separability and decision interpretation.

The present study does not require the input data normalization procedure. Little et al. [10] implemented the rescaling of feature values in the numerical range from −1 to 1, with the motivation of improving the SVM classification performance. Such data preprocessing, in our opinion, may cause some obstacles in data analysis. First, the rescaling or normalization is not robust for the data set of small size (the total number of voice records lower than 200 in the data set). Additional recruited voice records which exceed the current extreme of feature values would require another rescaling, such that the SVM classifier should be re-trained which consumes much more computation time. On the other hand, the physical magnitude information about the voice measurements would be lost after the data normalization. It is therefore not convenient for medical experts to use the data located around the discriminant boundary, for example the support vectors, as the important indicators for screening of pathological voice records. In addition, without the data normalization, the MAP classifier is able to achieve the overall accuracy of 91.8%, which is better than the previous related work (91.4% accuracy obtained by the SVM with ten normalized features) of Little et al. [10], and also comparable to the results (92.8% accuracy) performed by the SVM with bootstrap resampling of data in the work of Sakar and Kursun [3].

## Conclusion

Effective dysphonia detection provides more quantitative analysis of phonation disorders, toward better medical or behavioral treatments for speech improvement. In the present study, we studied the correlation matrix of the vocal measures that indicate the period, amplitude, and nonlinear perturbations in sustained vowel records. The dominant vocal measures of MDVP: F0, MDVP: Jitter (%), DFA, and Spread2 were selected by the SFS method, which could reduce the dimensions for pattern analysis. The nonparametric kernel density estimation method established the visible bivariate distributions of the KPCA-mapped feature densities. Based on the estimated feature densities, the MAP classifier was able to provide excellent classification performance, superior to that of the FLDA classifier and the SVM with RBF kernels. The experimental results demonstrated the merits of feature dimension reduction and kernel density modeling for vocal pattern analysis. The highest true positive rate (sensitivity) and minimal number of misclassified subjects also showed the effectiveness of the MAP classifier in the detection of phonation disorders. The relatively small size of phonation data limited the nonlinear classification capability of the SVM classifier. From the present work, we also conclude that the gender is not a sensitive factor for phonation disorders, and the PD patients with minimal functional disability are more likely to be incorrectly identified in the dysphonia detection. Noting that half of the SFS-selected vocal measures were generated by the nonlinear dynamics analysis tools, it is believed that the development of nonlinear vocal oscillation measurements has high potential in monitoring the progression of phonatory impairments in future studies.

## References

[1] Benninger MS, Ahuja AS, Gardner G, Grywalski C (1998) Assessing outcomes for dysphonic patients. Journal of Voice 12: 540–550. 9988041 10.1016/s0892-1997(98)80063-5

[2] Behroozmand R, Almasganj F (2007) Optimal selection of wavelet-packet-based features using genetic algorithm in pathological assessment of patients’ speech signal with unilateral vocal fold paralysis. Computers in Biology and Medicine 37: 474–485. 17034780 10.1016/j.compbiomed.2006.08.016

[3] Vaziri G, Almasganj F, Behroozmand R (2010) Pathological assessment of patients’ speech signals using nonlinear dynamical analysis. Computers in Biology and Medicine 40: 54–63. 19962694 10.1016/j.compbiomed.2009.10.011

[4] Pell MD, Cheang HS, Leonard CL (2006) The impact of Parkinson’s disease on vocal-prosodic communication from the perspective of listeners. Brain and Language 97: 123–134. 16226803 10.1016/j.bandl.2005.08.010

[5] Ho AK, Iansek R, Marigliani C, Bradshaw JL, Gates S (1998) Speech impairment in a large sample of patients with Parkinson’s disease. Behavioural Neurology 11: 131–137. 22387592

[6] Hartelius L, Svensson P (1994) Speech and swallowing symptoms associated with Parkinson’s disease and multiple sclerosis: a survey. Folia Phoniatr Logop 46: 9–17. 8162135 10.1159/000266286

[7] Holmes RJ, Oates JM, Phyland DJ, Hughes AJ (2000) Voice characteristics in the progression of Parkinson’s disease. International Journal of Language and Communication Disorders 35: 407–418. 10963022 10.1080/136828200410654

[8] Sewall GK, Jiang J, Ford CN (2006) Clinical evaluation of Parkinson’s-related dysphonia. Laryngoscope 116: 1740–1744. 17003722 10.1097/01.mlg.0000232537.58310.22

[9] Tsanas A, Little MA, McSharry PE, Ramig LO (2010) Accurate telemonitoring of Parkinson’s disease progression using noninvasive speech tests. IEEE Transactions on Biomedical Engineering 57: 884–893. 19932995 10.1109/TBME.2009.2036000

[10] Little MA, McSharry PE, Hunter EJ, Spielmanm J, Ramig LO (2009) Suitability of dysphonia measurements for telemonitoring of Parkinsons disease. IEEE Transactions on Biomedical Engineering 56: 1015–1022. 21399744 10.1109/TBME.2008.2005954 PMC3051371

[11] Alonso JB, de Leon J, Alonso I, Ferrer MA (2001) Automatic detection of pathologies in the voice by HOS based parameters. EURASIP Journal on Advances in Signal Processing 2001: 710108.

[12] Godino-Llorente J, Gomez-Vilda P (2004) Automatic detection of voice impairment by means of short-term cepstral parameters and neural network based detectors. IEEE Transactions on Biomedical Engineering 51: 380–384. 14765711 10.1109/TBME.2003.820386

[13] Little MA, McSharry PE, Roberts SJ, Costello DAE, Moroz IM (2007) Exploiting nonlinear recurrence and fractal scaling properties for voice disorder detection. Biomedical Engineering Online 6: 23. 17594480 10.1186/1475-925X-6-23 PMC1913514

[14] Zwirner P, Barnes GJ (1992) Vocal tract steadiness: a measure of phonatory and upper airway motor control during phonation in dysarthria. Journal of Speech and Hearing Research 35: 761–768. 1405531

[15] Hertrich I, Ackermann H (1995) Gender-specific vocal dysfunctions in Parkinson’s disease: electroglottographic and acoustic analyses. Annals of Otology, Rhinology, and Laryngology 104: 197–202. 10.1177/000348949510400304 7872602

[16] Goberman A, Coelho C, Robb M (2002) Phonatory characteristics of Parkinsonian speech before and after morning medication: the ON and OFF states. Journal of Communication Disorders 35: 217–239. 12064785 10.1016/s0021-9924(01)00072-7

[17] Goberman AM, Blomgren M (2003) Parkinsonian speech disfluencies: effects of l-dopa-related fluctuations. Journal of Fluency Disorders 28: 55–70. 12706913 10.1016/s0094-730x(03)00005-6

[18] Cnockaert L, Schoentgen J, Auzou P, Ozsancak C, Defebvre L, et al (2008) Low-frequency vocal modulations in vowels produced by Parkinsonian subjects. Speech Communication 50: 288–300.

[19] Rahn DA, Chou M, Jiang JJ, Zhang Y (2007) Phonatory impairment in Parkinson’s disease: evidence from nonlinear dynamic analysis and perturbation analysis. Journal of Voice 21: 64–71. 16377130 10.1016/j.jvoice.2005.08.011

[20] Bache K, Lichman M (2013). UCI machine learning repository. Available: http://archive.ics.uci.edu/ml. Accessed 2013 Dec 30.

[21] Hoehn MM, Yahr MD (1967) Parkinsonism: onset, progression, and mortality. Neurology 17: 427–442. 6067254 10.1212/wnl.17.5.427

[22] Guyon I, Elisseeff A (2003) An introduction to variable and feature selection. Journal of Machine Learning Research 3: 1157–1182.

[23] Duda RO, Hart PE, Stork DG (2001) Pattern Classification. New York, NY: Wiley, 2nd edition.

[24] Scholkopf B, Smola A, Muller KR (1998) Nonlinear component analysis as a kernel eigenvalue problem. Neural Computation 10: 1299–1319.

[25] Parzen E (1962) On estimation of a probability density function and mode. Annals of Mathematical Statistics 33: 1065–1076.

[26] Jain AK, Duin RPW, Mao JC (2000) Statistical pattern recognition: a review. IEEE Transactions on Pattern Analysis and Machine Intelligence 22: 4–37.

[27] Vapnik VN (1998) Statistical Learning Theory. New York, NY: Wiley.

[28] Wu Y, Krishnan S (2010) Statistical analysis of gait rhythm in patients with Parkinson’s disease. IEEE Transactions on Neural Systems and Rehabilitation Engineering 18: 150–158. 20650700 10.1109/TNSRE.2009.2033062

[29] Wu Y, Shi L (2011) Analysis of altered gait rhythm in amyotrophic lateral sclerosis based on nonparametric probability density function estimation. Medical Engineering and Physics 33: 347–355. 21130016 10.1016/j.medengphy.2010.10.023

[30] Wu Y, Cai S, Yang S, Zheng F, Xiang N (2013) Classification of knee joint vibration signals using bivariate feature distribution estimation and maximal posterior probability decision criterion. Entropy 15: 1375–1387.

[31] Sakar CO, Kursun O (2010) Telediagnosis of Parkinson’s disease using measurements of dysphonia. Journal of Medical Systems 34: 591–599. 20703913 10.1007/s10916-009-9272-y

